# Definitions of serious injury in long-term residential care: a systematic review protocol

**DOI:** 10.12688/hrbopenres.13705.1

**Published:** 2023-11-20

**Authors:** David Morrissey, Elizabeth A. O'Donnell, Laura Behan, Martin McMahon, Laura M. Keyes

**Affiliations:** 1 Health Information and Quality Authority (HIQA), Cork, T12 Y2XT, Ireland; 2 School of Nursing and Midwifery, Trinity College Dublin, Dublin, D02 PN40, Ireland

**Keywords:** Serious injury, serious incident, adverse event, reporting, residential care, nursing homes, care homes, systematic review.

## Abstract

**Background**: Evidence indicates that the reporting of serious injury in long-term residential care has increased substantially over the past decade. However, what constitutes a serious injury in residential care is poorly and inconsistently defined. This may result in incidences being unnecessarily reported as a serious injury. It is therefore, crucial to develop a consistent definition of serious injury to reduce reporting burden and to facilitate comparison between different residential care settings and across jurisdictions. This protocol describes the methods for a systematic review of existing definitions from the literature to inform the development of a consistent definition of serious injury in long-term residential care.

**Methods**: A wide range of published peer-reviewed and grey literature will be sought for this review, including guidance and policy documents. Searches will be conducted of databases including MEDLINE, CINAHL, SocINDEX, Academic Search Ultimate, and Westlaw International. Grey literature database searches will include Trip and Social Care Online. Country specific searches of government and health and social care websites will be conducted. Quality appraisal will be facilitated using the Quality Assessment for Diverse Studies (QuADS) tool and Tyndall’s checklist. The level of confidence in the findings will be assessed using the GRADE CERQual approach. A customised data extraction form will be used to extract data to reduce the risk of bias. Conceptual content analysis of data will facilitate identification of definitions of serious injury and their frequency within texts.

**Conclusions**: The findings will inform the development of a consistent definition of serious injury in long-term residential care that will reduce reporting burden, facilitate the accuracy of data collected and allow for comparison across jurisdictions. A more universal and consistent definition will enable regulators, policy makers, service providers and researchers to develop policy and practical interventions to prevent the occurrence of serious injury in long-term residential care.

## Introduction

The outcome of adverse events from unsafe care is likely one of the ten leading causes of death and disability in the world ^1^. Much of the patient safety literature to date has focused on adverse events in the context of acute settings ^2, 3^. The World Health Organisation have previously highlighted a lack of research examining adverse events in non-acute settings such as long-term residential care facilities ^4^. As noted by O’Regan *et al.*, (2022) targeted quality improvement initiatives in residential care facilities can be facilitated through the publication of adverse event reporting ^5^.

Across many jurisdictions such as Ireland, England, and Scotland, residential care providers have a legal obligation to report adverse events to the health and social care regulator ^6– 8^. These notifications describe significant events and incidents that are likely to have a negative impact on residents’ safety. These incidents may include, but are not limited to, the unexpected death of a resident, outbreak of a notifiable disease, serious injury to residents and allegations of abuse ^9^. The reporting of such incidents acts to facilitate transparency, future prevention and ongoing regulatory oversight.

Among countries with aging populations, one of the most challenging aspects of long-term residential care is in ensuring increasingly frail and vulnerable residents are protected wherever possible from serious injury. Incidents causing serious injury have a direct impact on the person that sustains the injury both in terms of the initial trauma and the potential for disability or loss of function as a consequence. Furthermore, serious injuries to residents place a strain on the limited resources available to health and social care systems ^10^. Data published by the regulator of social care in England has demonstrated that the reporting of serious injuries to residents in National Health Service (NHS) care homes increased year on year, almost doubling between 2011 and 2018 to 43,584 notifications ^11^. Likewise in Ireland, among the most frequently reported types of adverse events are notifications of serious injuries to residents in long-term residential care, particularly for older people ^5^. While public reporting and openly available notifications provide opportunities to analyse data pertaining to serious injuries in residential care facilities, it is necessary to review how serious injury is defined in long-term residential care, for example, to maximise the future utility of any serious injury data collected in this setting and to ensure time is not spent by service providers completing paperwork unnecessarily. 

A recent review has highlighted a continuing global inconsistency in the reporting of adverse events, both in terms of the approach implemented and the terminology used ^12^. For instance, in Ireland, the current regulations require service providers to provide notifications of any serious injuries that occur in residential care facilities, however, there is no legal definition of what constitutes ‘serious injury’. Guidance from the regulator for health and social care in Ireland defines a serious injury as follows: “Any bodily injury that involves a substantial risk of death, unconsciousness, extreme physical pain, protracted and obvious disfigurement, serious impairment of health or serious loss or impairment of the function of any bodily organ, for example fracture, burn, sprain/strain, vital organ trauma, a cut or bite resulting in an open wound, concussion, etc.” ^9^ This current definition is problematic due to the interpretative nature of the terms used. This is reflected by the substantial proportion of reported incidences that are unnecessarily labelled as “serious injury.” ^13^ A similar trend is apparent in other jurisdictions. Hence, the use of ambiguous and inconsistent language in describing serious injuries opens the door to over-reporting and/or inaccurate reporting of such events and makes it difficult to compare serious injury data between residential care settings and across jurisdictions. This makes it challenging for regulators, policymakers, service providers and researchers to develop policy and practical interventions to prevent the occurrence of serious injury in long-term residential care facilities with a negative impact on resident’s safety and well-being.

As noted by Hegarty *et al.* (2021) it is vital that the short-comings of serious incident reporting are swiftly addressed and this requires a renewed focus on specific categories of incidents including serious injury ^12^ For example, in Ireland, this has been identified as an area requiring review ^13^. Therefore, national and international evidence describing serious injury in long-term residential care will be analysed in this review.

The findings will assist the development of a consistent definition of serious injury in long-term residential care that will be applicable both nationally in Ireland, and internationally. In addition, the review findings will also identify definitions of serious injury used in regulatory frameworks. Expanding knowledge of what constitutes a serious injury in long-term residential care will be beneficial for regulators, service providers, policy makers and researchers to develop policy and practical interventions and strategies to prevent the occurrence of serious injury in long-term residential care.

## Research questions

1. What is a serious injury in long-term residential care?
2. What definitions of serious injury are used in regulatory frameworks?

The protocol will describe the:

▪ Process for conducting an extensive and systematic search for relevant articles, policy documents, guidelines and guidance documents.
▪ Eligibility criteria for inclusion in the review.
▪ Method for screening relevant articles and documents for inclusion.
▪ Methods of appraising the overall quality of individual studies, policy documents and guidelines.
▪ Methods of data extraction and data synthesis.

## Protocol

The protocol for this systematic review is registered at the PROSPERO database (CRD42022364546). This was registered on the 30/10/2022.

This protocol follows the PRISMA-P (Preferred Reporting Items for Systematic Reviews and Meta-Analysis Protocols; Moher *et al.*, 2015) guidelines. The PRISMA-P checklist can be found in Figshare ^14^.

## Criteria for inclusion

The phenomenon of interest is serious injury in long-term residential care.

There are no limits on inclusion in the review in terms of publication date, however, only articles or documents published in the English language will be included in this review.

Quantitative, qualitative and mixed methods research studies, literature reviews, policy, guidelines and guidance documents will be included in this review if they:

▪ Explicitly or implicitly define serious injury in long-term residential care.
▪ Identify criteria or elements of what constitutes a serious injury in long-term residential care.
▪ Identify definitions of serious injury that are used in regulatory frameworks.

Studies, guidelines, policy or guidance documents will be excluded if they:

▪ Explicitly or implicitly define serious injury in a context other than long-term residential care for instance, in the acute hospital setting, day care services, respite care, hospice care or home care.
▪ Defines serious incidents or identifies criteria of what constitutes a serious incident in long-term residential care that does not result in a serious injury to an individual in the context of direct physical harm, for example, breaches of data protection.
▪ The source relates to data obtained from editorials, opinion pieces and conference abstracts.

## Types of studies or documents to be included

There will be no specific limitations on the types of studies considered for inclusion in this review, therefore, quantitative, qualitative and mixed-methods studies of various designs will be eligible for inclusion. Given the topic of the research question and preliminary searches of the topic it is anticipated that findings from grey literature will contribute significantly to the findings of this review, for instance policy documents, guidance’s and guidelines identified by targeted searches of websites of regulatory organisations and Government agencies or organisations involved in the regulation of health and social care.

## Search methodology

The Population, Phenomena of Interest, Context (PICo) framework for developing a search strategy was used in this review ^13^ ( Table 1). Relevant search words were identified following a scoping search of literature using strings combined with the “AND” operator. An initial pilot search was conducted in the electronic database MEDLINE using the syntax outlined in Table 2. In addition, an initial search of the grey literature database Trip (version pro) was conducted using the syntax outlined in Table 3. The initial searches were used to refine the search strategy. Identification of known key articles within the search results confirmed the sensitivity of the search. Moreover, an information scientist was consulted to review and refine search strategies.

**Table 1.** PICo for the proposed refined research questions.

**Population**	Long-term residential care settings
**Phenomenon of interest**	Serious injury
**Context**	Any criteria used to define what constitutes a serious injury

**Table 2.** Search syntax for electronic database MEDLINE.

**Population**	**String 1**	'nursing home*’ or 'care home*’ or 'old age home*’or 'old age residen*’ or 'old people* home*’ or 'old people* residen*’ or LTCF or 'charitable hom*’ or 'charitable facilit*' or skilled nursing (facilit* or home* or residen*) OR (long term or long-term) (facilit* or home*) or retirement (facilit* or home*) or assisted living (facilit* or home* or communit*) or ‘residential facilit*’ or ‘residential care’ OR (MH "Nursing Homes+") or (MH "Skilled Nursing Facilities") or (MH "Residential Facilities+") OR (‘Children* home*’ or ‘Children residential facilit*’ or ‘Pediatric long-term care’ or ‘Pediatric long-term care facilit*’)
**Phenomenon** ** of interest**	**String 2**	‘serious OR critical OR severe OR significant OR substantial OR catastrophic OR adverse) (injur* OR harm* OR event* OR inciden*) OR ( ‘harmful event’ OR ‘patient harm’ OR ‘bodily harm’ OR ‘physical trauma’ OR ‘physical harm’ OR ‘adverse health’ OR ‘adverse outcome*’ OR ‘undesirable outcome*’ OR (MH "Drug-Related Side Effects and Adverse Reactions") OR (MH "Patient Safety")

**Table 3.** Search syntax for the database Trip (version pro).

Database	Search terms
Trip (version pro)	("residential care" OR "residential facility" OR "long-term care" OR 'nursing home*' OR 'care home*' OR 'old age home*') AND (“serious injury” OR “severe injury” OR “patient harm”)

## Information sources

A systematic literature search will be carried out using the following electronic databases: MEDLINE, CINAHL, SocINDEX with Full Text, Academic Search Ultimate, and Westlaw International. In addition, the reference list of the included full-text articles will be hand searched for relevant articles not retrieved in the original searches. Forward citation searching of included articles will also be conducted to identify any further articles for inclusion. The search terms for one electronic database (MEDLINE) are provided in Table 2.

Searches will also be conducted on established grey literature databases including Social Care Online and Trip (version pro) ( Table 3). The grey literature search will focus on specific jurisdictions with comparable healthcare systems, infrastructure and human development index scores, as previously used in research conducted by Hegarty *et al.*, (2021) ^12^. The following jurisdictions will be targeted: England, Wales, Scotland, Northern Ireland, Ireland, The Netherlands, Sweden, Denmark, the United States of America, Canada, Australia and New Zealand.

Targeted hand searches will also be carried out on the websites of identified organisations including regulatory and government agencies or organisations involved in the regulation of health and social care. For instance, the following websites will be examined: the World Health Organisation (WHO), The Social Care Institute for Excellence (SCIE), the National Institute for Health and Care Excellence (NICE), the Agency for Healthcare Research and Quality (AHRQ) and the National Quality Forum (NQF).

## Data (and software) availability

Microsoft Excel will be used in this review for screening articles. Mendeley 1.19 4 will be used for reference management.

## Screening

All references retrieved by the search terms will be imported into Microsoft Excel. All duplicates will be removed, and titles and abstracts of the references retrieved will be screened against the inclusion and exclusion criteria by two reviewers independently. Where disagreements arise, these will be resolved in the first instance, by discussion between the reviewers, and in the second instance through discussion with a third author where a final decision will be based on consensus. Following this, two reviewers will independently screen full-text studies for inclusion. Again, any disagreements that are not resolved following discussion will be adjudicated on by a third reviewer. The search strategy and study selection process will be reported in accordance with the PRISMA 2020 updated guideline for reporting systematic reviews statement. A PRISMA flow diagram will be generated.

## Quality appraisal

The review is expected to include a range of study designs, therefore, quality appraisal will be performed using the Quality Assessment for Diverse Studies (QuADS) tool and Tyndall’s checklist ^15, 16^. The synthesised findings of the included studies will be assessed for confidence using the Grading of Recommendations Assessment, Development and Evaluation Confidence in the Evidence from Reviews of Qualitative Research (GRADE CERQual) approach ^17^. Confidence of individual synthesised review findings will be based assessing the following four areas: the methodological limitations of the included studies, the relevance to the review question of the included studies, the coherence of the included studies, and the adequacy of data in the included studies. The overall assessment of confidence will be described under four levels: high, moderate, low or very low. Two reviewers will independently assess the strength of included articles. Any disagreements will be resolved through discussion, to achieve consensus. If required, a third reviewer will be consulted to make the final decision.

## Data extraction

Two reviewers will independently carry out data extraction for all articles included after the full text review. The reviewers will use a customised data extraction form ^18, 19^ to ensure consistency and reduce the risk of bias, thereby improving validity and reliability ^18^. Initially, the data extraction form will be trialled on three papers and iteratively modified to optimise for data extraction. Data to be extracted will include the authors, year, country, the aim of the study, the study design, setting, characteristics of the sample, the explicit or implicit definition of serious injury, and criteria or elements of what constitutes a serious injury (if applicable). In addition, the source and context of the definition will be extracted, for example, legislation, standards, guidance, empirical research or reviews. The extracted data will be compared, and any disagreements will be resolved by discussion between the reviewers. Any disagreements that are not resolved through discussion will be adjudicated by a third reviewer.

## Data synthesis

Conceptual content analysis will be applied to data to identify and determine the frequency of definitions of serious injury within texts. Content analysis is defined by Christie (2007) as “a tool to determine the presence of certain words or concepts within texts or sets of texts.” ^20^ Conceptual content analysis has been selected as the method of data analysis for this systematic review as Coe and Scacco (2017) indicate that it allows for quantitative analysis while remaining ‘close to the data’, to facilitate the acquisition of insight into complex human concepts and language ^21^.

Two researchers will independently code for specific words, phrases or sentences that define a serious injury. Words that explicitly state a definition of serious injury and words that imply a definition of a serious injury will be coded and extracted to a Microsoft Word document. Irrelevant text will be ignored when coding ^22^. The frequency of definitions of serious injury will be determined in general and then also in relation to regulation of residential care. Validity is ensured when coders are consistent in their use of codes. Intercoder reliability refers to the extent to which more than one coder independently codes in the same way as another coder. It is commonly used in content analysis and has been introduced as a measure for improving the approach's reliability ^23^. Therefore, both coders will independently code data in a pilot test. Actual coding will only commence after both coders achieve an intercoder reliability of 0.8. This Kappa statistic will be used to determine the consistency of coding between the two coders to ensure reliability, aligned with Sabharwal *et al.* (2018) ^24^.

The results will be reported in an organised and concise summary in the main body of the text ^25^, to define serious injury in general and in the context of regulation of residential care. Visual representations will also be presented illustrating the frequency of occurrence of definitions. Therefore, this approach is objective and systematic, as recommended by Bloor and Wood (2006) ^26^. In addition, the quality of data underpinning these results will be explicitly stated in the results section.

This systematic review will ensure scientific rigour by ensuring the validity and reliability of findings and by generating new insights, as recommended by Krippendorff (2006) to improve understanding of how serious injury is defined in general in the literature and also in relation to regulation in residential care ^27^.

## Dissemination of information

This systematic review will be submitted to a relevant peer review academic journal for publication. The findings from the review will be presented at relevant national and international conferences. Conference abstracts arising from the systematic review may also be published in peer-review journals. Furthermore, the review findings will be disseminated to regulators in the Health Information and Quality Authority (HIQA) in Ireland and other regulators in Europe *via* the Supervision and Regulation Innovation Network for Care (SINC). The findings of the review will also be presented to relevant government organisations and health and social care organisations both in Ireland and internationally. 

## Study status

Database searches using the search terms outlined in Table 2 have commenced.

## Strengths and limitations

To the best of the author’s knowledge this review will be the first review to identify definitions and descriptions of serious injury used in regulatory frameworks. This review will support the development of a definition of serious injury which will be beneficial to ensure consistency in use of terminology across multiple disciplines and jurisdictions. This will enable stakeholders including regulators, policy makers, service providers and researchers to develop policy and practical interventions and strategies to prevent the occurrence of serious injury in long-term residential care.

The comprehensive nature of the literature search outlined to include research studies and guidance and policy documents will ensure a broad spectrum of perspectives are included, increasing generalisability of findings. The narrow focus in terms of setting, *i.e.* long-term residential care, will promote specificity of findings. Moreover, taking a systematic and objective approach to data synthesis by applying conceptual content analysis will facilitate the acquisition of insight into complex human concepts and language of what constitutes serious injury in long-term residential care, aligned with Coe & Scacco (2017) ^20^.

The main limitation of this work is the decision to limit the grey literature search to jurisdictions with comparable healthcare systems, infrastructure and human development index scores. It is, therefore, possible that relevant documents or studies conducted in developing countries may be missed. However, including literature, particularly, policy and guidance documents from across international jurisdictions can hinder comparisons given the varied unique health and social care contexts. It is also challenging to implement given the large number of jurisdictions globally. In addition, this review may be limited by the restriction to only include literature published in the English language. However, a reliance on language translation software was deemed inappropriate given the intricacies of accurately identifying definitions of serious injury definitions in this review. 

## Conclusion

This protocol describes the systematic and objective methods that will be implemented in this review in relation to the search strategy, screening, quality appraisal, data extraction and data synthesis to address the research question: what is a serious injury in long-term residential care? Specifically, this review will assist in the development of a definition of serious injury in long-term residential care in general and also definitions of serious injury used in regulatory frameworks. It is envisaged that a consistent definition of serious injury will lead to more accurate notifications of such events to the regulator and facilitate the transfer of knowledge across stakeholders more easily. Importantly, a consistent definition of serious injury in long-term residential care will enable regulators, policy makers, service providers and researchers to develop policy and practical interventions to prevent the occurrence of serious injury in long-term residential care, and to improve health outcomes for residents.

## Data Availability

No data are associated with this article. Figshare: Data extraction form.docx. https://doi.org/10.6084/m9.figshare.21524787 ^19^ This project contains the following extended data: Data extraction form.docx The data extraction table provides for the extraction of data for this systematic review. It includes general information to be extracted from included studies and documents as well as specific data, relating to the explicit or implicit definition of serious injury, in addition to, criteria or elements of what constitutes a serious injury (if applicable). Figshare: PRISMA-P checklist for “Definitions of serious injury in long-term residential care: a systematic review protocol”. https://doi.org/10.6084/m9.figshare.21524853 ^14^ Data are available under the terms of the Creative Commons Attribution 4.0 International license (CC-BY 4.0).
