# Supplementary material for: Definitions of serious injury in long-term residential care: a systematic review protocol
Source: HRB Open Res. 2023 Nov 20;6:66. [Version 1] doi: 10.12688/hrbopenres.13705.1 (PMC10879755; doi:10.12688/hrbopenres.13705.1)
Supplement: Peer review history

# Reviewer report: Reviewer response for version 1

**DOI:** 10.21956/hrbopenres.14990.r37991

**Published:** 2024-05-13

**Authors:** Caroline Dalton

**Affiliations:** 1 University College Cork, Cork, County Cork, Ireland

Thank you for affording me the opportunity to review this protocol, related to the issue of serious injury in residential care homes. This is an interesting article, focusing specifically on how serious injuries are defined in residential care homes. The authors contend that the use of ambiguous terminology in defining serious injury, can have an impact on the reporting of these events in long term residential care, making it challenging to address the impact of serious injuries on residents in these services. Therefore, this is a contemporary issue, which needs to be addressed.

 Overall, the protocol is well developed and clearly written. Please find below some comments to enhance the quality and rigour of the review.  

 Major points which need to be addressed to enhance the quality of the protocol include:

 ·         Provide a short, succinct overarching aim for the protocol prior to the individual research questions.

 ·         The search string under “Population” in Table 2 clearly outlines the diverse range of   residential care homes to be included in the systematic review. However, providing a broad definition of residential care homes and making it clear that both adult and children residential care homes will be included, will further enhance the clarity of the protocol.

 The search methodology is clearly delineated with the use of appropriate criteria such as PICo, to ensure the validity and rigour of the review. A minor recommendation would be to clarify that the PICo will be used at the start of the “Criteria for inclusion” section.

 The research team have indicated that they intend to use Microsoft Excel to screen the studies. There are several forms of software available such as Covidence, that the team might consider using as they provide a more streamlined process for screening, which may make the review process less cumbersome for the team.

Is the study design appropriate for the research question?

Yes

Is the rationale for, and objectives of, the study clearly described?

Yes

Are sufficient details of the methods provided to allow replication by others?

Yes

Are the datasets clearly presented in a useable and accessible format?

Not applicable

Reviewer Expertise:

Nursing education, qualitative research, end-of-life care

I confirm that I have read this submission and believe that I have an appropriate level of expertise to confirm that it is of an acceptable scientific standard.

# Reviewer report: Reviewer response for version 1

**DOI:** 10.21956/hrbopenres.14990.r37993

**Published:** 2024-02-20

**Authors:** Mohamad Saab

**Affiliations:** 1 Catherine McAuley School of Nursing and Midwifery, College of Medicine and Health, University College Cork, Cork, County Cork, Ireland

Thank you for inviting me to review this protocol. Overall, it is well written. Below are my comments to improve the readability of the protocol and rigour of the intended review:

 ABSTRACT

- Avoid conjunctive adverbs in abstracts “however” “therefore”…
- See PRISMA extension for abstracts of systematic reviews to ensure that all elements of the review are being reported.

INTRODUCTION

- The use of “the” is redundant in several places for example “the findings will assist the development of” should read like “findings will assist in the development of”, “the review findings” (no need for “the”)…
- A single statement of the aim is needed before the research questions.
- The fact that the protocol is registered in PROSPERO is a strength. I query, however, the recency of this review given that the protocol was registered 16 months ago. Have there been any changes made to the protocol since?

CRITERIA FOR INCLUSION

- It is unclear which framework is underpinning the criteria for inclusion. PICO? PIO? PEO? PCC? Etc. This must be made explicit and appropriate resource cited. Inclusion and exclusion criteria should then be reorganised accordingly.
- I strongly encourage the authors to avoid the “English” database limit unless there is a strong reason to do so.  

SEARCH METHODOLOGY

- I now see that PICo will be used. Perhaps use the same framework to rework the criteria for inclusion.
- Not sure if Table 1 adds anything not already covered under CRITERIA FOR INCLUSION.

DATA (AND SOFTWARE) AVAILABILITY

- I query whether Microsoft Excel is the best software to screen studies. I recommend using Covidence (if funding is available) as this is often the best and most user-friendly tool/online software to deduplicate records and conduct blinded screening at title, abstract, and full text screening stages and also extract data. If funding is not available for a Covidence license, I recommend the free online software Rayyan.

STRENGTHS AND LIMITATIONS

- “To the best of the author’s knowledge” should read “To the best of the authors’ knowledge” since there is more than one author on this paper.
- I see that you justified the English limiter here.

Is the study design appropriate for the research question?

Yes

Is the rationale for, and objectives of, the study clearly described?

Partly

Are sufficient details of the methods provided to allow replication by others?

Yes

Are the datasets clearly presented in a useable and accessible format?

Yes

Reviewer Expertise:

oncology, men's health, patient safety, nursing education, systematic reviews

I confirm that I have read this submission and believe that I have an appropriate level of expertise to confirm that it is of an acceptable scientific standard.

# Reviewer report: Reviewer response for version 1

**DOI:** 10.21956/hrbopenres.14990.r37888

**Published:** 2024-02-07

**Authors:** Linda Birt

**Affiliations:** 1 INTO University of East Anglia, Norwich, England, UK

Thank you for the opportunity to review this literature review protocol designed to lead to a better definition of what constitutes a serious injury in care homes.

 Two suggestions to make clearer to reader what the search includes is to state focus on residents and not staff and make clear what types of residential setting will be included. Adding this to text will help even though evident in search terms

 A little pedantic but in the first sentence of introduction is the word ‘likely’ needed? Does the reference does not state whether unsafe it is or is not one of 10 leading causes of death?

Is the study design appropriate for the research question?

Yes

Is the rationale for, and objectives of, the study clearly described?

Yes

Are sufficient details of the methods provided to allow replication by others?

Yes

Are the datasets clearly presented in a useable and accessible format?

Not applicable

Reviewer Expertise:

Older people,  palliative care, qualitative methods

I confirm that I have read this submission and believe that I have an appropriate level of expertise to confirm that it is of an acceptable scientific standard.
